# Identification of Patients at Risk of Obstructive Sleep Apnea in Dental Settings

**DOI:** 10.7759/cureus.44646

**Published:** 2023-09-04

**Authors:** Mohammed A Sindi, Mohammed Mirdad, Maisa Al-Sebaei, Mohamed Bamashmous

**Affiliations:** 1 General Dentistry, King Abdulaziz University, Jeddah, SAU; 2 General Dentistry, Ministry of Health, Jeddah, SAU; 3 Oral and Maxillofacial Surgery, King Abdulaziz University, Jeddah, SAU; 4 Dental Public Health, King Abdulaziz University, Jeddah, SAU; 5 Orthodontic and Dentofacial Orthopedics, Boston University, Boston, USA

**Keywords:** sleep medicine, prevalence, early detection, berlin questionnaire, obstructive sleep apnea

## Abstract

Objectives

This study aims to assess the risk of obstructive sleep apnea (OSA) in the Saudi Arabian population at a dental hospital using the Berlin Questionnaire (BQ) and investigate the association of gender, age, neck circumference, systolic blood pressure (SBP), diastolic blood pressure (DBP), and smoking habits with the risk of OSA.

Methodology

Participants were recruited through random selection from walk-in patients aged between 18 and 80 years. BQ was used to screen for OSA. In addition, age, gender, smoking habits, neck circumference, and blood pressure were collected. Data were analyzed using descriptive statistics, Student's t-test, and chi-square test.

Results

In this study, 55 participants were screened for OSA using BQ. Of the participants, 44 (80%) were considered to be at low risk of OSA. Age, neck circumference, BMI, SBP, and DBP were statistically significantly associated with high risk of OSA (*P* < 0.05). Age and neck circumference were found to be statistically significant predictors of OSA, even after controlling for gender and smoking status (*P* < 0.05).

Conclusions

BQ is a reliable tool for assessing the risk of OSA in the Saudi Arabian population. Age, neck circumference, BMI, SBP, and DBP are all significant factors of OSA, while age and neck circumference are significant predictors of OSA. Dental practitioners can play a valuable role in the early detection and referral of patients at high risk of OSA.

## Introduction

Obstructive sleep apnea (OSA) is a prevalent sleep disorder that affects nearly one billion individuals worldwide [1]. This disorder is characterized by recurrent episodes of airway collapse, manifesting as obstructive apneas or hypopneas, wherein respiratory effort decreases by 90% or more and 30% or more, respectively, lasting for 10 seconds or longer [2]. Several factors have been implicated in the development and susceptibility to OSA, including reduced expansion forces of pharyngeal dilator muscles, excessive weight, hypertension, smoking, and increased neck circumference [3-6]. Common signs and symptoms of OSA include episodes of apnea, snoring, arousal, and daytime sleepiness [3]. The gold standard for diagnosing OSA is polysomnography (PSG), a comprehensive sleep test performed in specialized sleep centers that monitors neurophysiological, cardiopulmonary, and other physiological parameters overnight [7]. However, PSG is costly and time-consuming and requires a team of professionals.

Dental healthcare workers play a vital role in OSA management, from early detection and referral to active treatment and follow-up. Certain dental and facial features have been associated with an increased risk of OSA, such as increased lower facial height, maxillary retrognathism, mandibular retrognathism, elongated soft palate, and enlarged tongue [8]. Dentists and their teams can identify these abnormalities and refer patients accordingly, while dental specialists, including prosthodontists, orthodontists, and oral and maxillofacial surgeons, can provide treatment options such as mandibular advancement devices, uvulopalatopharyngoplasty (UPPP), OSA-Herbst, and continuous positive airway pressure (CPAP) devices, all of which have demonstrated positive impacts on patients with diagnosed OSA [9].

The early detection and prompt referral of high-risk OSA patients to specialized sleep centers can lead to substantial cost savings, amounting to tens to hundreds of billions of dollars [10]. Simple and user-friendly questionnaires have proven effective in the early detection of patients at risk of OSA, even when administered by untrained personnel. One such questionnaire is the Berlin Questionnaire (BQ), developed during the Conference on Sleep in Primary Care in Berlin, Germany, in 1996, and subsequently published by Netzer et al. [11]. The BQ consists of 10 subjective questions divided into three categories, and a body mass index (BMI) score. Each question is assigned points based on its most extreme answer, with categories 1 and 2 requiring a score of 2 points to be considered positive. Notably, in category 2, the last question (Q9) is applicable only if the preceding question (Q8) is answered affirmatively, contributing to the overall positivity of the category. For category 3, a positive response to its single dichotomous question or a BMI score greater than 30 counts it as a positive category. Participants with one or no positive categories are considered at low risk of OSA, while those with two or three positive categories indicate a high risk [11].

Despite the significance of the BQ and its potential as a screening tool, there is a notable dearth of studies in Saudi Arabia examining the risk of OSA using the BQ and its correlation with local factors. Alahmari et al. reported a high risk of OSA in 29% of truck drivers surveyed in the middle and eastern regions of Saudi Arabia using the BQ [12]. In contrast, Al-Dekhel and Banabilh found a lower prevalence of high-risk OSA (8.2%) among surveyed students and workers in the middle-northern regions [13]. Additionally, Al-Jewair et al. identified a significantly higher risk of OSA in male patients from Eastern Saudi Arabia (78.3%) compared to females (21.7%) using the BQ [14]. Obesity was found to be a significant factor, with obese patients having a 10-fold increased likelihood of being at a high risk of OSA compared to nonobese individuals. Notably, tongue indentations and tonsil grades III and IV exhibited a significant correlation with the risk of OSA, emphasizing the crucial role of dentists in recognizing signs and symptoms of OSA [14].

Therefore, the aim of this study is to assess patients who may be at risk of OSA using the BQ and investigate the association and implications of gender, age, neck circumference, systolic blood pressure (SBP), diastolic blood pressure (DBP), and smoking habits concerning the risk of OSA. By exploring these factors, we aim to enhance the understanding of OSA in the Saudi Arabian population and contribute to the development of effective strategies for its diagnosis and management.

## Materials and methods

Ethical approval

This study protocol received ethical approval from the Research Ethics Committee at the Faculty of Dentistry, King Abdulaziz University (REC-KAUFD), under approval number 043-15, and was conducted at King Abdulaziz University Dental Hospital (KAUDH), Jeddah, Saudi Arabia.

Participant recruitment

The study participants were recruited through random selection from walk-in patients who were aged between 18 and 80 years, visited the screening clinic at KAUDH between November 2016 and April 2017, and expressed their willingness to provide informed consent. To ensure privacy and confidentiality, no personal information was collected, and access to the collected data was restricted to the authors responsible for statistical analysis.

Data collection

The original BQ was used to screen for OSA. The BQ can be accessed on the website of the American Thoracic Society [15]. In addition to completing the BQ, the following data were collected: age, gender, smoking habits, neck circumference (cm), and blood pressure (mmHg). The practitioners were first given a lecture about OSA, trained on the BQ, and calibrated in correctly utilizing the tools (i.e., blood pressure monitor and measuring the neck circumference with a measuring tape). They collected the additional data under the supervision of the primary investigator to ensure a complete understanding of the process.

Data management and analysis

The recorded blood pressure was categorized into three groups (normal, elevated, and stage I and above) based on the 2017 classification by the American Heart Association and American College of Cardiology [16]. BMI was calculated by dividing each participant’s weight in kilograms by their height in meters squared. Participants with a BMI of 30 or above were grouped together.

Microsoft Excel 2021 (Microsoft Corporation, Redmond, WA, USA) was used to tabulate the data and define the groups. For data coding, statistical analysis, and table export, we used IBM SPSS Statistics for Windows, Version 25.0 (IBM Corp., Armonk, NY, USA).

Descriptive statistics were used to define the characteristics of our sample, including the mean (SD), percentages, and frequencies. The study employed a student's t-test and chi-square test to explore the association between BQ risk assessment and the predefined factors. A *P*-value of <0.05 was regarded as statistically significant.

## Results

In this cross-sectional study, 55 participants (age [mean ± SD] = 34.62 ± 14.9 years) consented to participate. The majority of the participants were female (34, 61.8%), nonsmokers (41, 74.5%), and had a neck circumference (mean ± SD) of 35.8 ± 5.9 cm. According to the BQ, 44 (80%) participants were considered to be at low risk of OSA. Additional descriptive statistics about the demographics can be seen in Tables 1-2.

**Table 1:** Descriptive statistics for demographics and health variables. SD, standard deviation; BMI, body mass index

Parameters (*n* = 55)	Minimum	Maximum	Mean	SD
Age (years)	18	78	34.62	14.9
BMI (kg/m^2^)	15.24	45.73	25.42	5.9
Neck circumference (cm)	25.00	47.00	35.80	5.3
Systolic blood pressure (mmHg)	98	168	128.09	16.2
Diastolic blood pressure (mmHg)	28	103	78.98	13.2
Berlin score	0	3	0.73	1.0

**Table 2:** Descriptive statistics for gender, smoking, and Berlin score category.

Parameter		Frequency (*n* = 55)	Percentage (%)
Gender	Male	21	38.2
	Female	34	61.8
Do you smoke?	No	41	74.5
	Yes	14	25.5
Berlin score category	Low risk	44	80
	High risk	11	20

Of the 55 participants, 13 (23.6%) had a positive response to category 1, 11 (20%) had a positive response to category 2, and 16 (29.1%) had a positive response to category 3.

· For category 1, 2 participants (15.4% of those who had a positive response) scored the maximum of 6 points. The mean score for category 1 positive participants, excluding those with scores less than 2, was 3.57 points, with a median and mode of 3.

· For category 2, 2 participants (18.2% of those who had a positive response) scored the maximum of 3 points. The mean score for category 2 positive participants, excluding those with scores less than 2, was 2.18 points, with a median and mode of 2.

· For those who had a positive response to category 3, 75% (12) were due to a BMI of greater than 30, and one-third of those additionally had hypertension. The mean score for category 3 positive participants was 1.13 points, with a median and mode of 1.

Descriptive statistics for all the questionnaire items and each Berlin category can be viewed in Table 3.

**Table 3:** Descriptive statistics for all the questionnaire items and each Berlin category.

Parameter		Frequency (*n* = 55)	Percentage (%)
1. Do you snore?	a. Yes	17	30.9
	b. No	38	69.1
2. Your snoring is: (Skip if no)	a. Slightly louder than breathing	9	45.0
	b. As loud as talking	6	30.0
	c. Louder than talking	5	25.0
	Skip if no	35	
3. How often do you snore? (Skip if no)	a. Nearly every day	4	22.2
	b. 3-4 times a week	7	38.9
	c. 1-2 times a week	3	16.7
	d. 1-2 times a month	4	22.2
	Skip if no	37	
4. Has your snoring ever bothered other people? (Skip if no)	a. Yes	11	55
	b. No	4	20
	c. Don’t know	5	25
	Skip if no	35	
5. Has anyone noticed that you stop breathing during your sleep? (Skip if no)	a. Nearly every day	1	9.1
	b. 3-4 times a week	2	18.2
	c. 1-2 times a week	3	27.3
	d. 1-2 times a month	5	45.5
	Skip if no	44	
6. How often do you feel tired or fatigued after your sleep?	a. Nearly every day	3	5.5
	b. 3-4 times a week	16	29.1
	c. 1-2 times a week	20	36.4
	d. 1-2 times a month	3	5.5
	e. Never or nearly never	13	23.6
7. During your waking time, do you feel tired, fatigued, or not up to par?	a. Nearly every day	2	3.6
	b. 3-4 times a week	11	20.0
	c. 1-2 times a week	15	27.3
	d. 1-2 times a month	5	9.1
	e. Never or nearly never	22	40.0
8. Have you ever nodded off or fallen asleep while driving a vehicle? if yes, answer (9).	a. Yes	7	12.7
	b. No	48	87.3
9. How often does this occur?	c. 1-2 times a week	2	28.6
	d. 1-2 times a month	4	57.1
	e. Never or nearly never	1	14.3
10. Do you have high blood pressure?	a. Yes	9	16.4
	b. No	42	76.4
	c. Don’t know	4	7.3
Summary			
Berlin Category 1	Negative	42	76.4
	Positive	13	23.6
Berlin Category 2	Negative	44	80.0
	Positive	11	20.0
Berlin Category 3	Negative	39	70.9
	Positive	16	29.1

A bivariate t-test and chi-square test showed that from our set of variables, both age and neck circumference were found to be statistically significant using Welch’s t-test (*P* < 0.05), while the BMI category, SBP category, and DBP category were found to be statistically significant using the chi-square test (*P* < 0.05). Additional details about both tests can be seen in Table 4.

**Table 4:** Bivariate t-tests and chi-square tests. ^a^Significant using Welch's t-test for *P* < 0.05. ^b^Significant using a chi-square test for *P* < 0.05. ^c^The total count of analyzed responses is 54, not 55, due to the corruption of some data related to a single participant who could not be recalled. SD, standard deviation

Demographics		Mean ± SD/Frequency (%)	Berlin score category, Mean ± SD/Frequency (%)			*P*-value
			Low risk of OSA, 44 (80%)		High risk of OSA, 11( 20%)	
Age (years)		34.62 ± 14.85	30.23 ± 9.55		52.18 ± 19.35	<0.001^a^
Neck circumference (cm)		35.80 ± 5.32	34.30 ± 3.87		41.82 ± 6.21	<0.001^a^
Gender	Male	21 (38.2%)		14 (66.7%)	7 (33.3%)	0.052
	Female	34 (61.8%)		30 (88.2%)	4 (11.8%)	
BMI category	Underweight	6 (10.9%)		6 (100%)	0 (0%)	0.001^b^
	Normal	22 (40%)		22 (100%)	0 (0%)	
	Overweight and obese	27 (49.1%)		16 (59.3%)	11 (40.7%)	
Systolic blood pressure category^c^	Normal	20 (37%)		20 (100%)	0 (0%)	0.007^b^
	Pre-high and high	34 (63%)		24 (70.6%)	10 (29.4%)	
Diastolic blood pressure category^c^	Low	2 (3.7%)		2 (100%)	0 (0%)	0.029^b^
	Normal	29 (53.7%)		27 (93.1%)	2 (6.9%)	
	Pre-high and high	23 (42.6%)		15 (65.2%)	8 (34.8%)	

A logistic regression model was used to predict the risk of having OSA. The predictors were age, gender, smoking status, and neck circumference. The results showed that the global model was statistically significant (*P* < 0.05). Age and neck circumference were statistically significant predictors of OSA, even after controlling for gender and smoking status (*P* < 0.05) (Table 5).

**Table 5:** Logistic regression model. ^*^Statistically significant for *P* < 0.05. CI, confidence interval

Variable	Odds ratio	95% CI for odds		*P*-value
		Lower	Upper	
Age (years)	1.078	1.016	1.144	0.013*
Gender	1.188	0.096	14.761	0.894
Do you smoke?	3.777	0.360	39.647	0.268
Neck circumference (cm)	1.320	1.027	1.697	0.030*

## Discussion

This paper investigates the risk of OSA in dental clinic patients based on the validated BQ questionnaire and addresses the correlation between the risk and other factors, such as gender, smoking status, neck circumference, BMI category, SBP, and DBP readings.

In our paper, we found that 36 (20%) patients were at high risk of OSA according to BQ. Our findings indicate higher percentages compared to those reported by Al-Saqqaf et al., who identified rates of 14.9% among sickle-cell anemia patients and 16.7% among their control patients, with no statistically significant difference between the two groups [17]. Similarly, Ahmad et al. reported a prevalence of 12.9% among patients attending family medicine clinics [18]. However, our findings reveal lower percentages when compared to those reported by several authors. For example, Al-Jahdali reported rates ranging from 34% to 37% in patients with end-stage renal disease undergoing dialysis and also noted a significant association between OSA risk and afternoon and evening dialysis shifts [19]. Alruwaili et al. documented a high risk of 33.4% among females and 31.1% among males in a study encompassing 2095 participants from the general population [20]. Bahammam et al. reported a prevalence of 33.3% among 578 middle-aged men [21]. Furthermore, Alahmari et al. identified a risk of 29% among 338 male truck drivers [12]. While most studies estimating OSA risk using the BQ were conducted in areas of Saudi Arabia other than the western region where this study was conducted, it is noteworthy that studies conducted in the western region, particularly in the city of Jeddah, generally report lower risk percentages compared to studies in other regions [17,18].

We found age to have a statistically significant correlation with the risk of OSA. This comes in agreement with many locoregional and international published literature [19,20,22,23]. Additionally, we found neck circumference to have a statistically significant correlation with the risk of OSA. This, also, goes in line with many previously published papers [14,17,19,22,24]. BMI is another factor that we found to have a significant statistical correlation and that has been linked many times to OSA risk [17,18,22,24,25]. Finally, we found both SBP and DBP readings to have a statistically significant correlation with the risk of OSA, and we found all the patients with normal SBP to be at a low risk of OSA. The link between OSA risk and blood pressure, both SBP and DBP, has been suggested and studied for a long period, and although local studies looking at this link are scarce, we found many to agree with our findings [11,17,25-27].

Overall, there are many similarities between our findings and both locoregional and international findings in terms of risk percentages and significant associations. This comes after the fact that data collection and reporting of data used in our paper was done by general dental practitioners who received no special courses or training in sleep medicine and disorders. This shows that dentists can play a major role in the early detection and referral of patients who may be at risk of developing OSA. Additionally, their work can be deemed reliable, although moving toward investigative studies with a bigger sample size may prove to be useful in further backing this statement. Furthermore, dentists, both general practitioners and specialists, can assist in monitoring, treating, and following up on OSA patients. Seeing how OSA is a very common syndrome that constitutes a large financial burden worldwide [10], the use of dental hospitals, dental clinics, primary dental care centers, and community dental centers can help alleviate the weight of this condition and improve the quality of life worldwide.

Nonetheless, there are some limitations encountered while performing the study. The sample size of our study was 55, and while this number was statistically sufficient for us to perform all necessary tests reliably, a large sample size would have given a more generalized look and would have allowed a more in-depth statistical analysis. The profile of our sample was another limitation, as including a wider variety from locations other than dental walk-in clinics would have benefited the variety and reliability of our data. Additionally, the scarcity of local papers that look at the issue as a whole and at each factor independently was a hurdle in designing the study and its aspects. Finally, as our sample was recruited from walk-in clinics, our participants may have been exposed to multiple types of bias.

## Conclusions

In this cross-sectional study, we found BQ to be a reliable tool in assessing the risk of OSA in our population. We also found our local population to be similar to other locoregional and global populations. Based on our data, we found age, neck circumference, BMI, SBP, and DBP to compose a statistically significant correlation with the risk of OSA. Moreover, we found age and neck circumference to be statistically significant predictors of high OSA risk. Our outcomes show the ability of dental practitioners to reliably assist in the early detection and referral of patients at high risk of OSA to specialized sleep centers.
